# Design of a Fuel Explosion-Based Chameleon-Like Soft Robot Aided by the Comprehensive Dynamic Model

**DOI:** 10.34133/cbsystems.0010

**Published:** 2023-03-14

**Authors:** Haiqin Zhou, Shunze Cao, Shuailong Zhang, Fenggang Li, Nan Ma

**Affiliations:** ^1^ Department of Mechanical Engineering, Beijing Institute of Technology, Beijing 100081, China.; ^2^ Department of Engineering Mechanics, Tsinghua University, Beijing, China.; ^3^ School of Mechatronical Engineering and Beijing Advanced Innovation Center for Intelligent Robots and Systems, Beijing Institute of Technology, Beijing 100081, China.; ^4^ Department of Engineering of Lancaster University, Lancaster LA1 4YW, UK.

## Abstract

Soft robotics have advantages over the traditional rigid ones to achieve the bending motion but face with challenges to realize the rapid and long-distance linear motion due to the lack of a suitable actuation system. In this paper, a new explosion-based soft robot is proposed to generate the axial fast extension by the explosion pressure. To support and predict the performance of this explosion-based soft robot, a novel dynamic model is developed by considering the change of working fluid (molecular numbers) and some unavoidable and influential factors in the combustion process. Then, based on the physical prototype, a set of experiments is conducted to test the performance of the explosion-based soft robot in performing the axial extensions, as well as to validate the model proposed in this article. It is found that the novel explosion-based soft robot can achieve rapid axial extension by the developed explosion-based actuation system. The explosion-based soft robot can achieve 41-mm displacement at a fuel mass of 180 mg. In addition, the proposed dynamic model can be validated with an average error of 1.5%. The proposed approach in this study provides a promising solution for future high-power density explosion-based soft robots.

## Introduction

In recent years, different kinds of soft robots have been developed to satisfy the requirements of the varying applications [1–4]. These soft material-based robotic systems have many advantages over the traditional rigid ones, such as the improved range of motion, better adaptability in unstructured areas, reduced cost, and lower weight. The hyperelastic materials (e.g., elastomers) are normally adopted to support the motion of the soft robotic systems. For example, a wide range of soft robotics have been developed in recent years to mimic the motion of the nature animals (e.g., Multigait soft robot [5] and Elephant’s trunk manipulator [6]), aiming to conduct the tasks in the unstructured environment and terrain exploration by their multiple degree of freedom bending capability. However, their capability for the long-distance and high-speed linear motion is rather constrained by their structure (e.g., constructed by multiple bending units) and actuation system (e.g., motor or pressurized air). Therefore, novel soft robots that can achieve the long-distance and rapid linear reaching are desired to develop the robotic system with advanced functions (e.g., the prey strategy of chameleon with an extendable tongue).

Also relevant to the actuation, different actuation methods have been studied over the past decades [7–10], including the electroactive polymer actuators [11–14], shape memory alloy (SMA) actuators [15–17], and pneumatic actuators [18–21]. For example, the electroactive polymer actuators can reduce the energy consumption and improve the expansion efficiencies. The SMA actuators can reduce the weight and dimension of the system. The pneumatic actuation can provide high actuating force. For the abovementioned actuators, due to either the bulk dimension or the slow response, they are not suitable to be combined with the soft robots to develop the advanced extendable robotic system, where the quick response, compact dimension, and powerful output are required. Promisingly, the explosion-based actuator can self-regulate the actuation pressure using the chemical decomposition of hydride in a closed space, where the high force and linear output motion can be achieved [22,23]. This actuator has the high force to weight ratio due to its simple structure and powerful output, where the complex transmission mechanism, energy accumulation mechanism, and energy release trigger mechanism can be omitted, which is the great advantage to reduce the overall weight of the system. Because of the abovementioned advantages of the explosion-based actuation system, it is selected as the actuation system to be combined with the developed linear motion soft robot to achieve the fast and long-distance motion.

Another challenge is how to design the soft robots that can be powered by the explosion to achieve the linear motion, as the explosive process coupled with the energy conversion processes (e.g., chemical dynamics, gas-phase dynamics, and fluid dynamic processes), which are critical to the successful working of system. To solve this problem, the special design principles are proposed by the researcher. For example, Rus and Tolley [24] presented an untethered soft-bodied robot using a combination of pneumatic and explosive actuators to execute directional jumping maneuvers. Shepherd et al. [5] demonstrated the rapid actuation of pneu-nets using a chemical reaction to generate explosive bursts of pressure. However, to the best knowledge of the authors, no soft robots powered by the explosion can realize the long-distance and high-speed linear motion.

Conventionally, the deformation generated by the explosion-driven soft robot was normally measured by experiments [22,25] with little theoretical analysis, since modeling is difficult due to the highly complex combustion process. However, modeling is necessary for the development of explosion-driven soft robots since it can reduce cost and optimize the geometrical parameters in the design, as well as to predict the deformation in reality. Recently, Bartlett et al. [22] used the Otto cycle as a thermodynamic model to predict the combustion process of an explosion-driven soft robot. However, the Otto cycle assumes that the working fluid is an ideal gas. But actually, there are some differences between the actual combustion process with the ideal process (e.g., working fluid, specific heat, and the number of molecules). So, the Otto cycle cannot accurately describe the combustion process and predict the energy produced by combustion. To the best knowledge of the authors, currently, there is no feasible model for the reliable analysis of the combustion process and also the deformation process of explosion-based soft robot that is caused by the combustion pressure.

**Fig. 1.**
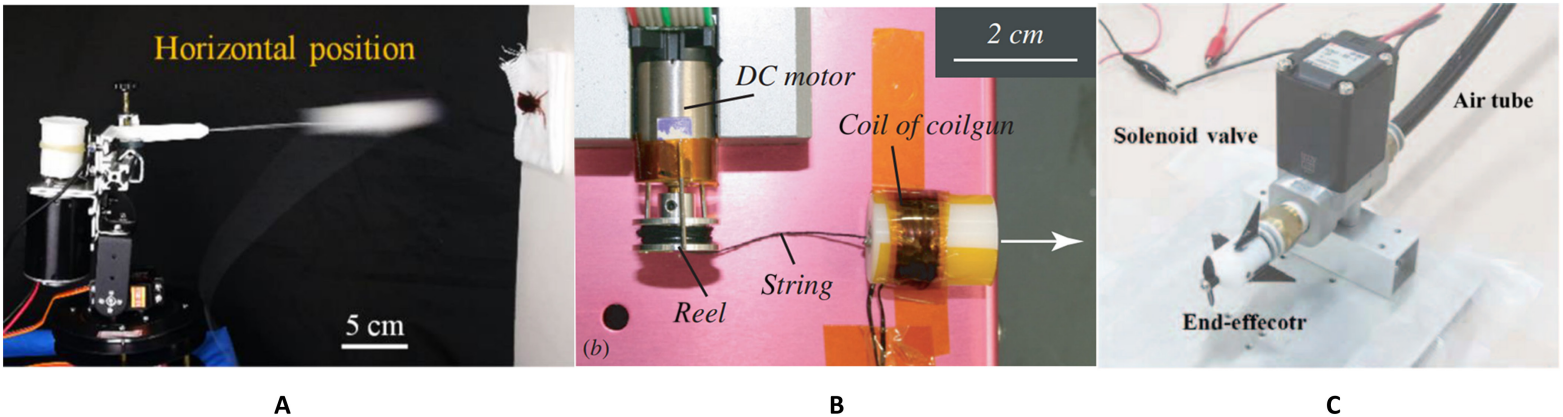
Examples of the shooting manipulator that imitate the function of the chameleon tongue. (A) Pressurized air-powered shooting system [19]. (B) Electromagnetic-powered shooting system [20]. (C) Shooting system with the passive propeller [21].

To address the aforementioned challenges of the soft robots to achieve the long-distance and high-speed linear motion, a novel explosion-based soft robot is proposed in this article. To support and predict the performance of the explosion-based soft robot, a new detailed dynamic model, which can be used to quantitatively predict the energy generated by the explosion and predict the deformation of the soft robot, is constructed. Further, the change of working fluid (molecular numbers) and some unavoidable and influential factors (e.g., heat transfer loss, specific heat, and incomplete combustion) are also calculated. At last, the performance of the explosion-based soft robot to achieve the axial extension is tested, which is further used to verify the dynamic model developed in this paper. The results show that the developed model can be validated by the experimental results, which can provide an efficient way to guide the structure design of the system in the initial stage (Fig. 1).

## Conceptional Design of the Novel Explosion-Based Soft Robot

**Fig. 2.**
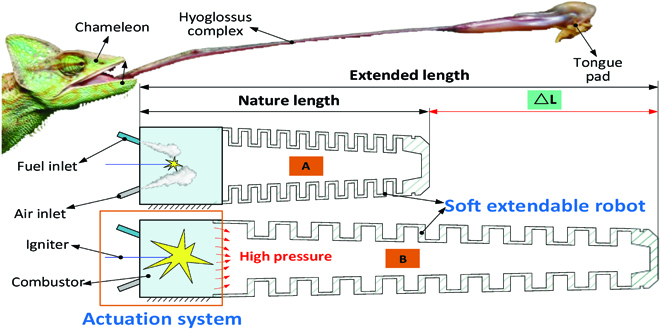
Concept design of the novel explosion-based soft robot to achieve the long-distance and high-speed linear motion.

In this section, a novel chameleon-like soft robot is introduced (see Fig. 2), which can achieve the large elongation by a novel actuation method (i.e., fuel explosion) and its special structure design (i.e., modular elastic unit). Specifically, the novel explosion-based actuation method is adopted to generate the required combustion pressure by fuel explosion in the designed combustor. Then, the elongation of the modular elastic units can be generated by the combustion pressure. With such a generic concept, the chameleon-like soft robot actuated by the fuel explosion system can enhance the motility of the robotic system.

### Concept design of the chameleon-like soft robot system

The chameleon tongue has several special functions that attract lots of attention. First, it can be rapidly thrown out (acceleration: around 10*g*) to prey the bugs. Second, the tongue can be extracted 10 times than its natural length to throw the tongue pad far away. Further, the sticky tongue pad can firmly hold the prey. As the high pressurized air can be generated in an instant by fuel explosion, to use this power carefully and in high efficiency, we selected the mechanism that can achieve the linear motion. With the powerful actuation system, the soft linear mechanism can achieve the fast-shooting function, enabling the system to achieve the function of chameleon tongue.

In this paper, we are focusing on the expansion/retraction geometry of the chameleon tongue, aiming to propose a new actuation method to bury the current challenges. Based on the motion principle of the chameleon tongue, many robots have been developed to imitate the function of the chameleon tongue. For example, a chameleon tongue-inspired shooting manipulator, which is actuated by the high pressurized air, was developed to achieve fast and accurate preying at a large distance [26]. Further, another chameleon tongue-inspired robot, which is actuated by the coilgun–elastomer system, was studied to throw out the magnet tongue pad [27]. Another shooting manipulator, which has a passive propeller at the tip of the end-effector to suppress the disturbance of the external forces, was developed to achieve 10 m reaching [28]. However, the aforementioned shooting robots normally either need the bulk actuation system (e.g., air compressor) or face the limited power of the actuation system (e.g., electromagnetic coil) to achieve long-distance reaching.

To address the aforementioned challenges on chameleon-like soft robot system, a new accordion structure is developed to achieve the linear movement of the chameleon tongue. Further, aided with the new explosion-based actuation system, the fast-shooting linear movement of accordion structure can be generated by the pressurized air, while the contraction movement will be achieved by the elastic potential energy. The overall design principle can be seen in Fig. 2. The actuation system includes fuel inlet, air inlet, the combustor, the igniter, and an enclosed space for the explosion of the mixed fuel and air. The soft extendable robot will be fabricated by the hyperelastic material, which can realize the elongation by the combustion pressure generated in the actuation system. In the initial stage, the chameleon-like robot is at its natural state without high pressure inside, as shown in Fig. 2A. After fuel explosion, the elongation of the chameleon-like soft robot will be generated by the combustion pressure, as shown in Fig. 2B.

### Structure design of the chameleon-like soft robot system

**Fig. 3.**
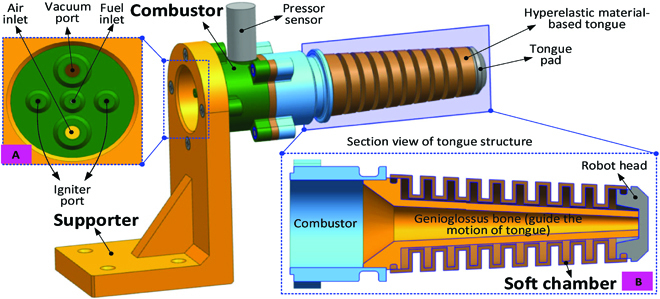
Structure of the chameleon-like soft robot. (A) Interface panel for the combustor. (B) Structure of the soft robot for achieving the extension function.

To materialize this idea, the structure of the chameleon-like soft robot system is designed to use fuel explosion for actuating the movement of the soft robot, as shown in Fig. 3. The structure design of the robot system is composed of the following 3 items: (a) the supporter, which provides the connection interface with the experimental table; (b) actuation system, which is constituted by the enclosed space and the necessary ports (e.g., air inlet, fuel inlet, and igniter); (c) modular elastic unit, which is made with hyperelastic material.

The actuation system is an important component for actuating the chameleon robot as achieving explosion inside the chamber is not a straightforward process due to the influence of the ratio of fuel and air. The fuel and air inlets are designed to ensure that the fuel and air quickly flow into the combustion chamber, as shown in Fig. 3A. In addition, 2 igniter ports are designed to be connected with the positive and ground poles of the voltage transformer.

As the chameleon-like robot will be fabricated by the hyperelastic material (e.g., silicone rubber) to achieve the large axial elongation, the downward radial deformation will happen by the weight of the robot. In order to keep the shape of the chameleon-like robot, the central genioglossus bone structure (with the angle θ) is designed to guide the motion direction of the soft robot, as shown in Fig. 3B.

**Fig. 4.**
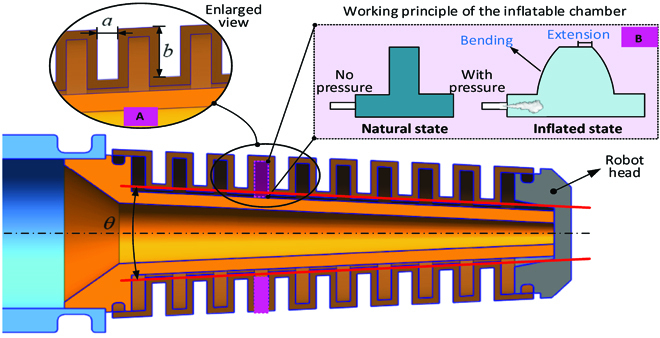
Detailed structure and working principle for achieving the extension function of the chameleon-like soft robot. (A) Enlarged view of the modular chamber. (B) Working principle of the soft robot for achieving the extension function.

To achieve the large axial elongation of the chameleon-like robot, the modular elastic unit is designed, as shown in Fig. 4A. Because of the special structure design of the modular chamber, the large axial elongation can be easily obtained with fuel explosion in the combustor. Specifically, the elongation of the chameleon-like robot is mainly generated by the following 2 components: one is axial extension; another one is bending of the soft robot, as shown in Fig. 4B.

### Working principle and control logic design

**Fig. 5.**
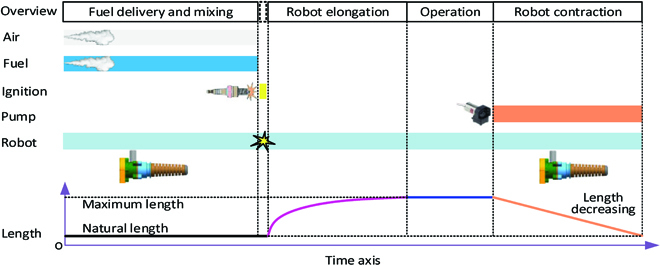
The working process and control logic of the chameleon-like soft robot.

Before the explosion occurs, 3 conditions must be met simultaneously. Fuel and air must exist in certain proportions, along with an ignition source [29,30]. Based on the concept design and the experimental experience of the chameleon-like robot, the working principle and control logic of the system can be described in Fig. 5.

The working principle of the chameleon-like soft robot can be described as the following steps: step 1, the air pump works and air flows into the combustion chamber after the intake valve opens; step 2, the valve of the fuel inlet opens and fuel flows into the chamber; step 3, the ignition system is working, leading to fuel explosion, and the elongation of the chameleon-like robot will be generated in this step; step 4, the air pump works again to extract the air in the chamber, achieving the chameleon-like robot to its natural length.

To achieve a successful explosion, the mass of fuel and air in the chamber needs to be accurately controlled. The fuel and air ratio will decide not only the combustion pressure generated by the explosion but also the successful explosion. In this study, the inlet valves of fuel and air are connected to the electric switches to control the mass of the fuel and air flowed into the combustor. The mass of fuel and air will be decided by the opening time of the corresponding electrical switches as the inlet valves fully open, and the fluid velocity is regarded as constant in this study. The ignition time in step 3 is decided by the fuel and air mixture process. In this study, the igniter is activated 3 s after the valve of fuel inlet is closed.

## Modeling of the Novel Explosion-Based Soft Robot

**Fig. 6.**
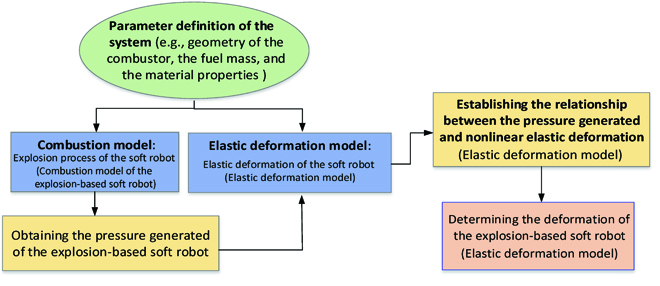
A flowchart to describe the overall dynamic model of the explosion-based soft robot.

Unlike the conventional approaches (e.g., Michael T. Tolley—Theory Method), a new dynamic model is proposed to investigate the relationship between the combustion pressure and the deformation of the soft robot based on the hyperelastic material property of silicone rubber. The proposed mathematical model can provide theoretical contributions to the deformation modeling of the explosion-based soft robot. The modeling starts from the Combustion model of the explosion-based soft robot section, where the combustion process is modeled to obtain the combustion pressure. Next, in the Elastic deformation model section, the elastic deformation model is analyzed to address the relationship between the combustion pressure and the deformation of the soft robot. A flowchart of the overall model is shown in Fig. 6.

The combustion model first considers the change of working fluid (molecular numbers) during the combustion process. The combustion model is also amended by considering some unavoidable and influential factors (e.g., heat transfer loss, specific heat, and incomplete combustion). With the established model, the combustion pressure can be calculated under the given conditions. In the elastic deformation model, our approach is based on assuming that an actuator consists of multiple segments (mimicking chameleon tongue), where each different segment undergoes some combination of extension and bending. Then, the relationship between the pressure generated by combustion and nonlinear elastic deformation (extend and bending) is built. By establishing the relationship between the combustion pressure and nonlinear elastic deformation, the energy generated by the explosion and the deformation of the soft robot can be predicted after giving the fuel and air mass.

### Combustion model of the explosion-based soft robot

**Fig. 7.**
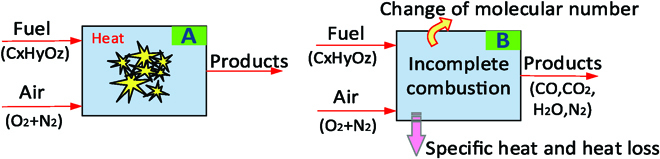
Schematic of the combustion model. (A) Otto cycle model used in the conventional approaches [22]. (B) Novel combustion model in this study.

In the proposed soft robot, an explosive actuator is adopted to replace the conventional actuation. The new design brings the advantages of the high load capacity, fast response, and high energy utilization rate. However, it also brings the challenges in specifying the energy generated by the explosion-based soft robot. The Otto cycle is an ideal combustion model, which was used as a thermodynamic model in the conventional approaches, as shown in Fig. 7A. However, there are some differences between the actual combustion process and the ideal process. Thus, in order to quantitatively analyze the energy generated by the explosion, a dynamic model of an explosion-based soft robot that considers the change of working fluid (molecular numbers) during the combustion process is built at first, as shown in Fig. 7B. The model also fully investigates some unavoidable and influential factors (e.g., heat transfer loss, specific heat, and incomplete combustion) in the combustion process.

#### Working fluid

*(a) The air mass*

In the proposed combustion model, the decomposition of hydride happens in a closed space and the chemical energy is converted into heat energy instantly. The air mass at the complete combustion process can be calculated based on the chemical reaction. We assume the carbon mass is *g*_C_ (unit: kg), the hydrogen mass is *g*_H_ (unit: kg), and the oxygen mass is *g*_O_ (unit: kg) per kilogram of fuel, so we can gain.

$$g_{c}+g_{H}+g_{0}=1$$ (1)

If carbon is completely burned and transformed into carbon dioxide, hydrogen will combine with oxygen and turn into carbon dioxide. So, there are *g*_c_/12 mol of CO_2_ and *g*_H_/2 mol of H_2_O. The air mass can be established as follows.

$$L_{0}=\frac{1}{0.21}\left(\frac{g_{c}}{12}+\frac{g_{H}}{4}-\frac{g_{0}}{32}\right)$$ (2)

*(b) Number of molecules and the stoichiometric molecular coefficient*

Equation 3 shows the number of molecules before combustion in this model.

$$M_{1}={\mathrm{aL}}_{0}$$ (3)

where *a* is the excess air coefficient.

Equation 4 shows the number of molecules after combustion used in this model.

$$M_{2}={\mathrm{aL}}_{0}-0.21L_{0}+\frac{g_{c}}{12}+\frac{g_{H}}{2}$$ (4)

The stoichiometric molecular coefficient is shown below.

$$\mu_{0}=\frac{M_{2}}{M_{1}}$$ (5)

*(c) The molecular coefficient*

There is still some exhaust gas in the closed container at the end of each combustion cycle. We assume that the exhaust gas mass is *M_r_*, so the total mass before combustion is

$$M_{1}^{\prime }=M_{1}+M_{r}={\mathrm{aL}}_{0}+M_{r}$$ (6)

The total mass after combustion is

$$M_{1}^{\prime }=M_{2}+M_{r}$$ (7)

The exhaust gas coefficient is the ratio of the exhaust gas mass to the number of molecules before combustion. The exhaust gas coefficient used in this model is shown

$$\gamma =\frac{M_{r}}{M_{1}}=\frac{M_{r}}{{\mathrm{aL}}_{0}}$$ (8)

Thus, the molecular coefficient is shown below.

$$\mu_{1}=\frac{M_{2}^{\prime }}{M_{1}^{\prime }}=\frac{M_{2}+M_{r}}{{\mathrm{aL}}_{0}+M_{r}}=\frac{\mu_{0}+\gamma }{1+\gamma }$$ (9)

The Otto cycle was used as a thermodynamic model in the conventional approaches. The ideal Otto cycle assumes that the working fluid is an ideal gas. For the actual combustion process, the working fluid is fresh air before combustion and is combustion products after combustion, leading the molecular numbers to be different. In order to build an accurate model, a new combustion model considering the effect of changing working fluid during the combustion process is established first in this study.

#### Combustion process

The effect of changing working fluid during the combustion process is discussed in the Working fluid section. For some unavoidable and influential factors (e.g., heat transfer loss, specific heat, and incomplete combustion), we will discuss them in this section. In this study, the time when the soft robot starts to deform is defined as the end of combustion due to most of the fuel being burned at this time. So, the following can be obtained

$$\xi_{z}H_{u}={\mathrm{xH}}_{u}-Q_{w}$$ (10)

where *ξ_z_* is the heat utilization factor, *H_u_* is the low heat value, *x* is the heat release coefficient, and *Q_w_* is the heat transfer loss to the wall.

According to the energy conservation equation, the following can be obtained

$$\xi_{z}H_{u}=U_{z}-U_{c}+W_{\mathrm{cz}}$$ (11)

where *U* is the internal energy, *W* is the work, *z* is the end of the combustion, *c* is the start of the combustion, and *cz* is the combustion duration.

In this study, we assume that it is constant–volume combustion, so *W_cz_* = 0. Then

$$\begin{array}{c} \xi_{z}H_{u}=U_{z}-U_{c}\\ =\left(M_{2}+M_{r}\right)\times c_{v}^{\prime \prime }t_{z}-\left(M_{1}+M_{r}\right)\times c_{v}^{\prime }t_{c} \end{array}$$ (12)

where *t* is the temperature, $c_{v}^{\prime \prime }$ is the specific heat of combustion products, and $c_{v}^{\prime }$ is the specific heat of working fluid. The following can be obtained

$$\frac{\xi_{z}H_{u}}{\left(1+\gamma \right)M_{1}}+c_{v}^{\prime }t_{c}=\mu_{1}c_{v}^{\prime \prime }t_{z}$$ (13)

Equation 15 shows the pressure ratio used in this model.

$$\lambda_{p}=\mu_{1}\frac{t_{z}}{t_{c}}$$ (14)

Then, the peak combustion pressure at the end of combustion is

$$p_{z}=\lambda_{p}p_{c}\eta_{e}$$ (15)

where *p_c_* is the initial pressure and *η_e_* is the pressure utilization factor.

In this section, the effects of some unavoidable and influential factors (e.g., heat transfer loss, specific heat, and incomplete combustion) are discussed to improve the accuracy of the dynamic model. With the established model, the combustion pressure can be calculated under any given conditions.

### Elastic deformation model

**Fig. 8.**
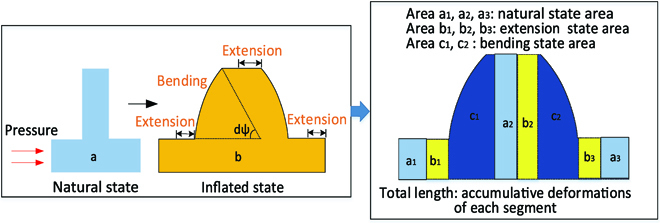
Schematic of the elastic deformation model of the explosion-based soft robot.

In this section, the relationship between the pressure generated by combustion and nonlinear elastic deformation will be built. The analytical model studies the elongation and bending of the modular units when the pressurized air is applied by the explosion actuation system. Our approach assumes that the soft robot consists of multiple segments (mimicking chameleon tongue), where each segment undergoes the combination of extension (area b_1_, b_2_, b_3_) and bending (area c_1_, c_2_), as shown in Fig. 8. The total length variation of the actuator is the accumulative deformations of each segment.

In the following, we first construct the strain energy expressions in the Strain energy function section. Then, to simplify the analytical modeling, we decouple the bending from the extended motion. We first introduce a model for soft robot that extends upon pressurization in the Modeling extension section, followed by a model that bends upon pressurization in the Modeling bending section.

#### Strain energy function

It is important to select the reasonable and practical strain energy density function to describe the mechanical property of a rubber-like material. In this section, we model the behavior of the soft robot powered by explosion with typical large deformation theories. Gent [31] proposed a new strain energy function for the nonlinear elastic behavior of rubber-like materials, and it is widely used. We adopt this model, and the strain energy function can be expressed as

$$W=\frac{\mu J_{m}}{2}\ln\left(1-\frac{I_{1}^{n}-3^{n}}{J_{m}}\right)$$ (16)

where *μ* is the shear modulus, *n* is the material parameter, and *J_m_* is the constant limiting value for *I*_1_ − 3.

Based on the elastic finite deformation theory, the left Cauchy–Green tensor can be denoted as **B** = **F** ∙ **F***^T^*, where **F** is the gradient of the deformation. By utilizing strain energy (Eq. 17), we can obtain the Cauchy stress tensor as shown below.

$$\sigma =-pI+2B\frac{\partial W}{\partial I_{1}}-2B\left(I_{1}-B\right)\frac{\partial W}{\partial I_{2}}$$ (17)

where *p* is the undetermined scalar function that justifies the incompressible internal constraint conditions.

#### Modeling extension

**Fig. 9.**
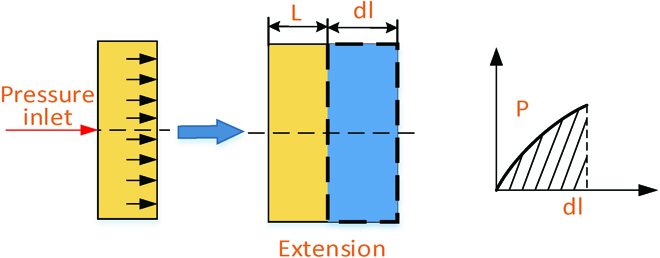
Schematic of the extension modeling of the explosion-based soft robot.

In this section, the relationship between the pressure generated by the combustion and extension deformation will be built. The analytical models for the explosive actuator that generates the extension of the soft robot will be presented. Our approach assumes that the tube retains its cylindrical shape upon pressurization because the elastomeric part of the actuator is of uniform stiffness, so the tube extends linearly with applied pressure, as shown in Fig. 9. The original length of the tube is *L*, and the added length is *dl*. As the pressure increases, the length of *dl* increases. The total extension deformation is the accumulative extent of each tube.

In this study, we consider the cylindrical rubber tube under the uniform pressure. (*R*, *Θ*, *Z*) and (*r*, *θ*, *z*) are the coordinates of the rubber tube before deformation and after deformation, respectively. *A* is the inner radius, *B* is the outer radius, and *L* is the length of the tube before deformation (*A* ≤ *R* ≤ *B*,0 ≤ *Θ* ≤ 2*π*,0 ≤ *Z* ≤ *L*). Then, the deformation pattern of the rubber tube can be shown as follows

$$r=f\left(R\right)\theta =f\left(\Theta \right)z=f\left(Z\right)$$ (18)

According to the incompressible condition

$$r=f\left(R\right)=\sqrt{a^{2}+\lambda_{z}^{-1}\left(R^{2}-A^{2}\right)}$$ (19)

The deformation gradient tensor **F** can be expressed as

$$F=\left|\begin{array}{ccc} \lambda_{1} & 0 & 0\\ 0 & \lambda_{2} & 0\\ 0 & 0 & \lambda_{3} \end{array}\right|=\left|\begin{array}{ccc} \frac{\mathrm{dr}}{\mathrm{dR}} & 0 & 0\\ 0 & \frac{r}{R} & 0\\ 0 & 0 & \lambda_{Z} \end{array}\right|$$ (20)

where *λ_z_* is the axial principal elongation ratio.

So, the principal stretch in the radial, circumferential, and axial direction of the cylinder membrane can be expressed as

$$\lambda_{1}=\frac{\mathrm{dr}}{\mathrm{dR}}={\left({\lambda \lambda }_{z}\right)}^{-1}\lambda_{2}=\frac{r}{R}=\lambda \lambda_{3}=\lambda_{z}$$ (21)

The left Cuachy–Green deformation tensor **B** can be defined as

$$B={\mathrm{FF}}^{T}=\left|\begin{array}{ccc} {\left({\lambda \lambda }_{z}\right)}^{-2} & 0 & 0\\ 0 & \lambda^{2} & 0\\ 0 & 0 & \lambda_{z}^{2} \end{array}\right|$$ (22)

So, the first invariants of the left Cuachy–Green deformation tensor **B** can be established as follows

$$I_{1}=\mathrm{tr}B={\left({\lambda \lambda }_{z}\right)}^{-2}+\lambda^{2}+\lambda_{z}^{2}$$ (23)

Substituting Eqs. 24 and 21 into Eq. 18

$$\left\{\begin{array}{c} \sigma_{\mathrm{rr}}=-p+2{\left({\lambda \lambda }_{z}\right)}^{-2}\frac{\partial W}{\partial I_{1}}\\ \sigma_{\theta \theta }=-p+2\lambda^{2}\frac{\partial W}{\partial I_{1}}\\ \sigma_{\mathrm{zz}}=-p+2\lambda_{z}^{2}\frac{\partial W}{\partial I_{1}} \end{array}\right.$$ (24)

For the cylinder rubber tube under internal pressure, the radical stress is zero outside of the rubber tube and the radical stress is equal to the internal pressure, which can be expressed as

$$\sigma_{\mathrm{rr}}\left(a\right)=-p\;\sigma_{\mathrm{rr}}\left(b\right)=0$$ (25)

where *a* = *f*(*A*), *b* = *f*(*B*).

From Eqs. 25 and 26, the following can be obtained

$$\begin{array}{l} \int_{-p}^{0}d\sigma_{\mathrm{rr}}=\int_{a}^{b}\frac{1}{r}\left(\sigma_{\theta \theta }-\sigma_{\mathrm{rr}}\right)\mathrm{dr}\\ =\int_{a}^{b}\frac{1}{r}\left(2\lambda^{2}\frac{\partial W}{\partial I_{1}}-2{\left({\lambda \lambda }_{z}\right)}^{-2}\frac{\partial W}{\partial I_{1}}\right)\mathrm{dr} \end{array}$$ (26)

By utilizing the expression *λ* = *r*/*R*, the following can be expressed

$$\mathrm{dr}=\frac{R}{1-\lambda^{2}\lambda_{z}}\mathrm{d\lambda}$$ (27)

Substituting Eq. 27 into Eq. 28, the following equation can be obtained

$$p=\int_{\lambda_{a}}^{\lambda_{b}}\frac{1}{\lambda }\frac{\partial W}{\partial I_{1}}\left[\lambda^{2}-{\left({\lambda \lambda }_{z}\right)}^{-2}\right]\frac{1}{1-\lambda^{2}\lambda_{z}}\mathrm{d\lambda}$$ (28)

where *λ_a_* = *a*/*A*, *λ_b_* = *b*/*B*.

We assume that rubber-like materials are incompressible. The following equations can be achieved

$$\left(\lambda^{2}-a^{2}\right)\lambda_{z}=R^{2}-A^{2}$$ (29)

Equation 30 can be transformed into

$$\lambda_{a}^{2}\lambda_{z}-1={\left(\varepsilon +1\right)}^{2}\left(\lambda_{a}^{2}\lambda_{z}-1\right)$$ (30)

Equation 31 can be transformed into

$$\lambda_{a}^{2}\lambda_{z}-1={\left(\varepsilon +1\right)}^{2}\left(\lambda_{a}^{2}\lambda_{z}-1\right)$$ (31)

where *ε* = (*B* − *A*)/*A*, 1 ≫ *ε* > 0.

So, the relationship between the pressure generated by combustion and nonlinear elastic deformation can be built. A simplified equation form can be expressed as

$$p=\frac{\mu {\mathrm{nJ}}_{m}}{J_{m}-\left(I_{1}^{n}-3^{n}\right)}I_{1}^{n-1}\left[\lambda^{2}-{\left({\lambda \lambda }_{z}\right)}^{-2}\right]\frac{\varepsilon }{\lambda^{2}\lambda_{z}}$$ (32)

In this section, the relationship between the pressure generated by combustion and extension deformation is built. With the established model, the extension deformation can be calculated under the given combustion pressure.

#### Modeling bending

Since the bending is caused by the moment generated by the combustion pressure acting on the soft robot, the relationship between the input pressure and the bending angle can be built. In this section, we present the bending model and built the relationship between the combustion pressure and bending deformation. First, we assume that the radial expansion and stress in the radial direction can be neglected. Furthermore, no twisting takes place since the soft robot has a symmetric arrangement of fibers, so the deformation gradient reduces to

$$F=\left|\begin{array}{ccc} \lambda_{z}{\left(\phi \right)}^{-1} & 0 & 0\\ 0 & 1 & 0\\ 0 & 0 & \lambda_{z}\left(\phi \right) \end{array}\right|$$ (33)

The soft robot bends due to the moment that is generated by the combustion pressure. *dφ* is the differential angle.

So, we can obtain the force due to the combustion pressure acting on an infinitesimal area of the actuator

$$\mathrm{df}=\mathrm{PdA}=2{\mathrm{PR}}_{i}\;\sin\mathrm{\phi dh}$$ (34)

where *P* is the combustion pressure in the actuator, *dh* is the differential height, and *h* = *R_i_* − *R_i_* cos *φ*.

Equation 35 can be transformed into

$$\mathrm{df}=\mathrm{PdA}=2{\mathrm{PR}}_{i}^{2}\sin^{2}\mathrm{\phi d\phi}$$ (35)

So, the moment generated by the combustion pressure acting on the soft robot can be expressed as

$$\begin{array}{c} M_{\mathrm{mon}}=\int_{0}^{2\mathrm{Ri}}\mathrm{df}\;\times \text{distance from neutral axis}\\ =2{\mathrm{PR}}_{i}^{2}\int_{0}^{\pi }\sin^{2}\text{ϕabs}\left(R_{i}\cos\bar{\phi }-R_{i}\cos\phi \right)\mathrm{d\phi} \end{array}$$ (36)

The opposing moment due to the stress in the material can be expressed as

$$M_{\mathrm{mar}}=2\int \lambda_{z}^{-1}\sigma_{\mathrm{zz}}R_{i}\left(R_{i}\cos\bar{\phi }-R_{i}\cos\phi \right)\mathrm{d\phi}$$ (37)

where *σ_zz_* is the axial stress in the outer anisotropic layer with shear modulus *μ_i_*

$$\sigma_{\mathrm{zz}}=\lambda_{z}^{2}\mu_{i}-\frac{\mu_{i}}{\lambda_{z}^{2}}$$ (38)

Now, solving *M*_mon_ = *M*_mar_ yields the relationship between combustion pressure and output bending angle.

$$P=\frac{2\int \lambda_{z}^{-1}\sigma_{\mathrm{zz}}R_{i}\left(R_{i}\cos\bar{\phi }-R_{i}\cos\phi \right)\mathrm{d\phi}}{2R_{i}^{2}\int_{0}^{\pi }\sin2\text{ϕabs}\left(R_{i}\cos\bar{\phi }-R_{i}\cos\phi \right)\mathrm{d\phi}}$$ (39)

In this section, the relationship between the combustion pressure and the bending angle is built. With the established model, the bending deformation can be calculated under the pressure generated by combustion conditions.

## Experimental Validation and Performance Study

In the Conceptional Design of the Novel Explosion-Based Soft Robot section, a novel kind of explosion actuated chameleon-like soft robot was developed, which was composed of the actuation system and soft extendable robot. In the Modeling of the Novel Explosion-Based Soft Robot section, a new kind of combustion model, which quantitatively studies the energy generated by the fuel combustion and predicts the deformation of the explosion-based soft robot, was established to study the dynamic characters of the developed soft robot. In this section, the ability of the explosion-based soft robot to an axial extension is tested in experiments. In order to validate the proposed dynamic model expressed by Eqs. 1 to 39, a set of experiments is conducted by increasing the fuel mass.

### Prototype and experimental setup

**Fig. 10.**
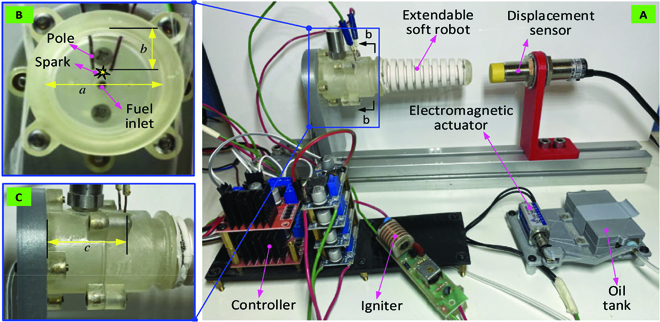
Experimental setup and detailed structure of the overall system (i.e., chameleon-like soft robot, combustion system, and control system). (A to C) Note: A commercial ignition system (fuel: C_4_H_10_, igniter: electric) and an extendable soft robot (material: silicone rubber) are adopted as an example to demonstrate the concept of this paper.

In this study, the explosion-based chameleon-like robot is powered by the explosion for the locomotion. The experimental setup includes the fabrication of the soft robot and combustor, fuel mass control system, ignition system, control system, host computer, and power supply, as shown in Fig. 10. To power the developed soft robot, liquid *n*-butane (C_4_H_10_) is used as the fuel source due to its commercial availability in portable packages (e.g., in cigarette lighter fuel sources) and its high energy density in liquid form (28 MJ/l). The fuel mass control system is composed of the commercial lighter and electromagnetic actuator (model: SDO-0520L, voltage: DC 6 to 24 V). The valve of the lighter is connected with the electromagnetic actuator for controlling its state. When the electromagnetic actuator is “ON” with the power, the valve will be opened, while when the electromagnetic actuator is “OFF” without the power, the valve will be closed. Thus, with the developed control system, the fuel mass can be accurately controlled by adjusting “ON” time of the valve. The ignition system, which is controlled by the actuation system, can generate the spark for the mixed fuel and air for the explosion. By regulating the ignition time, the 5-V voltage can be generated for the ignition system and then increased to 20,000 V for generating the spark.

The combustor, which is the chamber for the fuel and air mixture for the explosion, is designed in cylinder shape, as shown in Fig. 10 (i.e., B-B for the section view and c for the front view). The pole for generating the spark is located at the center of the chamber (i.e., location: a, 25 mm; b, 10 mm; c, 30 mm). The fuel inlet is located at the geometrical center of the bottom plate, which is connected by the flexible tube with the oil tank (a lighter). A groove feature is designed at the tip of the combustor, which is used to connect the soft robot. To capture the extended length of the developed chameleon-like robot, a displacement sensor is adopted (type: M18NPN).

To achieve the accurate fuel mass and ignite time control for the fuel explosion, the control algorithm is designed based on the structured LabView FPGA control system (FPGA type: NI-9694, LabView version: 2017). The operations are as follows: first, the fresh air flows into the combustor; second, the valve of the fuel inlet is open and the fuel flows into the chamber; third, the igniter is working to generate the spark; last, the large deformation of the chameleon-like robot will be generated by the explosion of the mixed gas. During the overall process, the displacement sensor is working to capture the position of the tip of the chameleon-like robot.

**Fig. 11.**
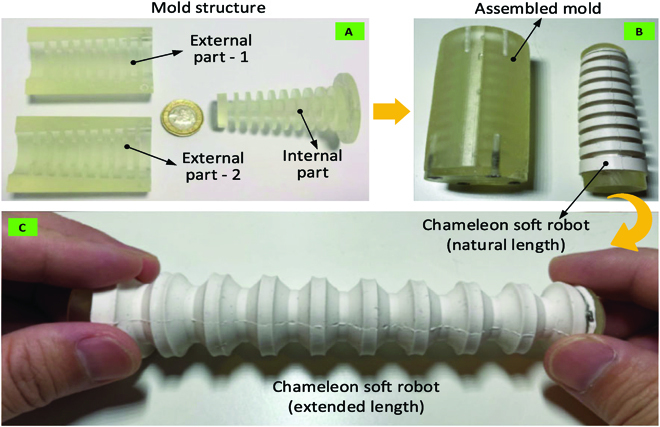
Process for fabricating the chameleon-like robot. (A) Mold to form the desired features of the chameleon soft robot. (B) Working principle of the mold and nature length of the chameleon-like robot. (C) Extended length of the chameleon-like robot.

To fabricate the chameleon-like robot that can have large extendable deformation, the silicon rubber is selected. First, the mold is designed to form the desired features of the chameleon-like robot, which is composed of 3 parts (i.e., internal and external parts, respectively), as shown in Fig. 11A. Second, these 3 parts can be assembled by the poisoning holes and screws to form the mold, as shown in Fig. 11B. With the sprue in the mold, the liquid silicone rubber can be delivered into the chamber. After the required cure time (around 16 h), the silicone rubber can be transferred from liquid state to solid state, as shown in Fig. 11B for the nature length of the chameleon-like robot. The extended chameleon-like robot is seen in Fig. 11C, where the large extendable ability can be achieved by adopting the developed structure.

### Explosion study of the combustor

#### Explosion range

**Fig. 12.**
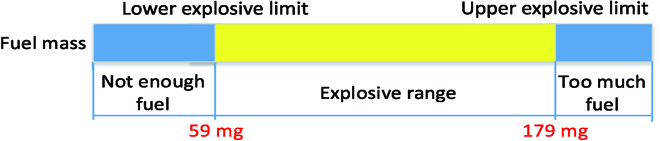
Explosion range for the given features of the combustor.

The successful implementation of a chameleon-like robot, which uses explosions from the fuel combustion for the high-speed linear motion, leads to great challenges. The high pressures generated from fuel combustion impose stress on the chameleon-like robot structure. The most challenging is achieving the combustion inside the combustion chamber, which is not a straightforward process due to the influence of the fuel and air mixture process on ignition and combustion. Before the explosion can occur, 3 conditions must be met simultaneously. Fuel and air must exist in certain proportions, along with an ignition source. The exact gas mix ratio that is required for an explosion depends on the type of fuel and the chamber volume. The minimum concentration of the fuel necessary to support its explosion in the air is defined as the lower explosive limit (LEL). Below this level, the mixture is too “lean” to burn. The maximum concentration of the fuel that will cause an explosion is defined as the upper explosive limit (UEL). Below this level, the mixture will burn, provided that oxygen is available, until the concentration increases to the UEL. The range between the LEL and UEL is known as the explosive range for the fuel, as shown in Fig. 12.

Before attaching the chameleon-like robot to the combustor, the explosion range is studied under different fuel mass conditions to find the suitable mass range of C_4_H_10_ for a better explosion. By gluing the transparent tape at the tip of the combustor, a closed space can be formed for the explosion. A set of experiments is carried out by increasing the mass of C_4_H_10_ by controlling the injection duration time in this study. The explosion process is assessed by the combustion flame captured by the high-speed camera, which was placed in front of the combustor. It is found that the fuel mass of LEL and UEL is 59 and 179 mg, respectively, in this study.

#### Explosion process

**Fig. 13.**
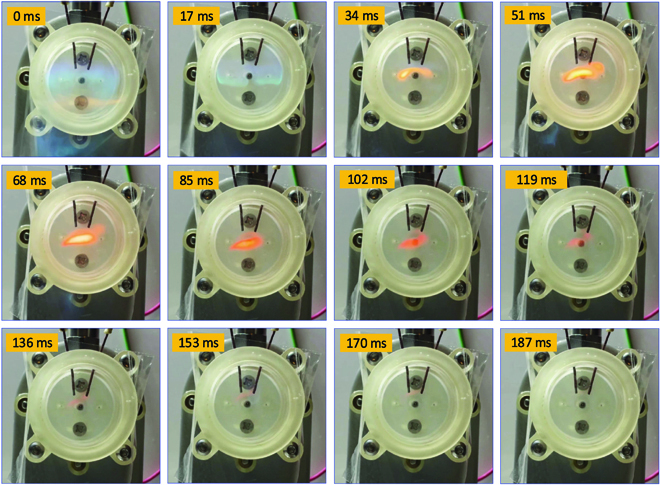
Explosion flame study of the combustor under the given fuel mass of C_4_H_10_. Note: To form the enclosed space, the transparent tape was glued at the tip of the combustor. The high-speed camera was placed in front to capture the explosion flame.

To better understand the explosion process, continuous flame images of C_4_H_10_ combustion with a fuel mass equal to 65 mg are shown in Fig. 13. The snapshots are selected with 17-ms intervals, and the time the flame appears is defined as the initial time of the explosion in this study. From the figures, we can see that in the first stage, the explosion first occurs near the ignition and the color of the flame is blue. The main reason is that at the beginning of the explosion, the air is sufficient and complete combustion occurs. As the combustion progresses, the air in the chamber decreases, which had an impact on the color of the flame. It turns yellow (at the time of 34 ms). The main reason is that the oxygen in the combustion chamber gradually decreases, and incomplete combustion occurs. Once the explosion is ended, there is no flame in the chamber, as shown in the last figure.

In this section, the experimental explosion range for C_4_H_10_ is conducted on the proposed explosion-based chameleon-like robot. By controlling the injection duration time, the fuel mass is increased and measured, respectively. It could be observed that the explosion mass range of C_4_H_10_ in this study is 59 to 179 mg. Then, the combustion flame with a fuel mass equal to 65 mg is studied, which is important for understanding the explosion process in the chamber.

#### Model validation for the explosion-based soft robot

As commented, a chameleon-like soft robot is capable of the axial extension by its special structure, which is actuated by fuel explosion. In this section, the performance of the explosion-based soft robot to the axial extension is tested with experiments, which is further used to check the correctness of the dynamic model developed in this paper.

**Fig. 14.**
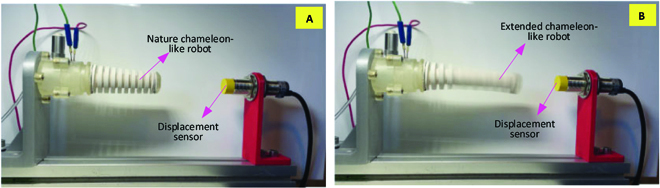
Dynamic response of the chameleon-like soft robot. (A) Nature chameleon-like soft robot in the initial stage. (B) Extended chameleon-like robot under the explosion actuation.

After completing the preparation process defined in the Prototype and experimental setup section for the explosion, the chameleon-like soft robot can be assembled on the combustor, as shown in Fig. 14A, the natural state of the robot. With the explosion power, the large material deformation of the silicone rubber will happen, enabling the chameleon-like soft robot to be extended along its axial direction, as shown in Fig. 14B. In the experiments, the displacement of the soft robot is captured by a displacement sensor installed on the front support. It can also be seen from Fig. 14B that the gravity has its effect in the movement of the robot, but this influence has its limitation as the elongation period is quite short, leading to the small deviation.

**Fig. 15.**
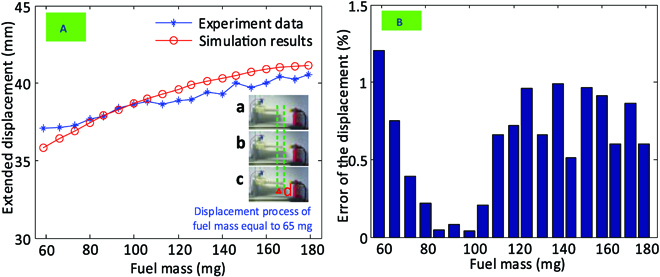
Comparison between theoretical calculations and experimental results of the displacement of the chameleon-like soft robot under different fuel masses. (A) Displacement variation of the chameleon-like soft robot. (B) Error of the displacement.

The dynamic model is of key importance to predict the output of the system (i.e., deformation of the chameleon-like soft robot) for the given inputs (i.e., fuel and air mass). In order to check the dynamic response of the chameleon-like soft robot under different fuel mass conditions, a set of experiments was performed with different fuel masses (i.e., from 59 to 179 mg with 6-mg interval), as shown in Fig. 15. Between each test, the preparation process defined in the Prototype and experimental setup section needs to be performed to ensure the accuracy of the experimental result. From the experimental results, it can be seen that the displacement of the explosion-based soft robot increases with the increase of the fuel mass. The explosion-based soft robot can achieve 41-mm displacement at a fuel mass of 180 mg. It can also be seen that the simulation results match well those from the experiments (average error: 1.5%) at the measured point. Under the 2 different fuel masses (i.e., 93 and 100mg), the maximum error between the simulation and experimental results is 0.8 and 0.5 mm, respectively. Hence, it can be concluded that the explosion-based soft robot is capable of axial extension by its special structure. The proposed model is accurate for predicting the displacement of an explosion-based soft robot.

In this section, the experimental explosion range for C_4_H_10_ is conducted first. It could be observed that the explosion mass range of C_4_H_10_ in this study is 59 to 179 mg. Then, the combustion flame with a fuel mass equal to 65 mg is studied, which is important for understanding the explosion process in the chamber. After studying the explosion process, the displacement of the explosion-based soft robot under different fuel masses is studied and the experimental results are used to verify the dynamic model developed in this paper. It could be observed that the proposed dynamic models have been proved accurate (average error: 1.5%).

## Conclusions

In this article, a novel explosion actuated chameleon-like soft robot is proposed, which uses explosion to achieve the axial extension. A theoretical model is proposed to investigate the dynamics of the explosion-based soft robot. The novelties can be summarized as follows:

• A novel kind of explosion actuated chameleon-like soft robot is developed. The ability to achieve the axial extension would allow the soft robots to realize long-distance and high-speed linear motion (e.g., prey motion), which greatly expands their capabilities for the new applications such as fast-speed long-distance prey and rescue. In addition, the novel actuation system developed in this paper can greatly improve the force/mass ratio of the system, which provides a promising way to improve the performance and reduce the overall weight of the next-generation soft robots.
• A detailed formulation and explanation of a novel dynamic model for the explosion-driven soft robots has been developed. The relationship between the pressure generated by combustion and nonlinear elastic deformation is built to predict the displacement of the explosion-based soft robot under different fuel masses. Then, the validation process was conducted based on the experimental results. It was found that the proposed dynamic models have been proved accurate (average error: 1.5%). The developed dynamic model provides an effective way to guide the structure and control algorithm design of the explosion-based soft robots. It also fills the gap for the explosion-based soft robots, where the exhaustive theoretical and methodological approach to theoretical modeling is highly needed.

## Data Availability

The data are freely available upon request.
